# Construction and *in vivo* characterization of an infectious clone of porcine circovirus type 3 from southwestern China

**DOI:** 10.1128/spectrum.03865-25

**Published:** 2026-04-13

**Authors:** Li-na Shao, Bao-ling Liu, Tong Xu, Han-yu Li, Lei Zhao, Jia-qi Duan, Li-shuang Deng, You-you Li, Le-yi Zhang, Shu-ying He, Yang-ming Dai, Xin Wu, Si-yuan Lai, Zhi-wen Xu, Ling Zhu

**Affiliations:** 1 College of Veterinary Medicine, Sichuan Agricultural University 506176 , Chengdu, China; 2 Sichuan Key Laboratory of Animal Epidemic Disease and Human Health, College of Veterinary Medicine, Sichuan Agricultural University 506176 , Chengdu, China; Changchun Veterinary Research Institute, Chinese Academy of Agricultural Sciences, Changchun, China

**Keywords:** porcine circovirus type 3 (PCV3), infectious clone, pathogenicity, tissue tropism, southwestern China

## Abstract

Porcine circovirus type 3 (PCV3) is an emerging viral pathogen associated with multi-systemic inflammation, porcine dermatitis and nephropathy syndrome, and reproductive failure, leading to substantial economic losses in the global swine industry. Although PCV3 has been widely detected in southwestern China, its pathogenic characteristics remain insufficiently understood. In this study, an infectious clone of the PCV3/SC/Sichuan-2023 strain was constructed using the pBluescript SK(+) backbone and subsequently employed to rescue the recombinant virus rPCV3. The recovered rPCV3 replicated efficiently *in vitro* and induced overt clinical manifestations and pathological lesions in piglets that closely recapitulated those observed under field conditions. Strong viral antigen signals were detected predominantly in the lungs and lymphoid tissues, confirming distinct tissue tropism. Similarly, rPCV3 infection elicited significant upregulation of pro-inflammatory cytokines, reflecting the immune dysregulation characteristic of natural infection. These results demonstrate that rPCV3 reproduces essential pathogenic features observed during experimental infection and aligns closely with reports from field strains. Furthermore, the established infectious clone provides a robust reverse-genetics platform for virus rescue, stable propagation in PK-15 cells, and comprehensive *in vitro* and *in vivo* characterization, facilitating future investigations into PCV3 pathogenesis and the development of effective control strategies in southwestern China.

**IMPORTANCE:**

Porcine circovirus type 3 (PCV3) is widely prevalent in pig populations; however, its biological characteristics and pathogenic mechanisms remain incompletely understood. In this study, we generated a full-length infectious clone of a field-derived PCV3 strain. We demonstrated that the rescued virus faithfully reproduces critical aspects of natural infection, including robust replication, distinct tissue tropism, and cytokine dysregulation in piglets. These findings provide valuable experimental tools and novel insights into PCV3 pathogenesis, supporting enhanced disease control strategies and future vaccine development.

## INTRODUCTION

Porcine circovirus type 3 (PCV3) is a small, single-stranded circular DNA virus belonging to the genus *Circovirus* and shares a similar genomic organization with PCV1 and PCV2. The PCV3 genome comprises three major open reading frames (ORFs): ORF1 encodes the replication-associated protein (Rep), ORF2 encodes the capsid protein (Cap), and ORF3 produces a protein whose function has not yet been fully characterized (1–3). Although PCV3 isolates show a high degree of genetic conservation, with more than 94% nucleotide identity, sequence variations are predominantly concentrated within the Cap gene, particularly at positions 8–10, 24, 27, 77, 137, and 150 (4). These mutations may affect antigenic diversity and host adaptation.

Since its initial identification in 2016, PCV3 has been reported across Asia, Europe, and the Americas. It has been associated with porcine dermatitis and nephropathy syndrome (PDNS), respiratory and reproductive disorders, and systemic inflammatory conditions (5–7). The virus affects pigs at all developmental stages; however, pregnant sows and suckling piglets appear to be particularly susceptible (8–10). PCV3 frequently co-infects with immunosuppressive pathogens, such as porcine reproductive and respiratory syndrome virus (PRRSV), which can exacerbate disease severity (11, 12). PCV3 has been detected in clinically healthy pigs, indicating that subclinical or latent infections may also occur (13, 14). Despite its broad geographic distribution and heterogeneous clinical presentation, the pathogenic mechanisms of PCV3 remain incompletely understood. This knowledge gap is attributable mainly to difficulties in isolating viruses using conventional cell culture systems and to the limited availability of infectious clones for experimental investigation.

Although recent reverse-genetics approaches have enabled the successful rescue of PCV3 (15, 16), the biological characteristics and virulence of field-derived strains remain insufficiently characterized, particularly in southwestern China. This region represents a major swine-producing area with a high prevalence of PCV3 but relatively limited experimental data on viral pathogenicity (17). To address these gaps, we constructed a full-length infectious clone of the PCV3/SC/Sichuan-2023 strain using the pBluescript SK(+) vector and successfully rescued a viable recombinant virus (rPCV3). The recovered virus replicated efficiently *in vitro* and induced characteristic clinical signs, histopathological lesions, and cytokine dysregulation in piglets. This study describes the first infectious clone-derived PCV3 strain from southwestern China with experimentally confirmed pathogenicity, providing a robust platform for future investigations into PCV3 virulence mechanisms and host–pathogen interactions. The Sichuan isolate showed a close genetic relationship with PCV3 strains previously reported in Korea, suggesting a potential transboundary transmission event linked to international breeding stock trade.

## MATERIALS AND METHODS

### Collection of specimens

An outbreak primarily affecting weaned piglets was detected at a commercial swine farm in Sichuan Province. The first cases emerged in September 2023, and the outbreak persisted until November 2023. Twenty clinical specimens, including kidneys, spleens, hearts, brains, livers, lungs, lymph nodes, and serum, were collected from affected animals. Clinically, the piglets exhibited rough hair coats and multiple papular and macular cutaneous lesions (Fig. S1). All specimens were immediately transported on ice and stored at −80°C until further processing.

### Extraction, amplification, and cloning of the PCV3 genome

Tissue samples were homogenized in sterile phosphate-buffered saline (PBS) and centrifuged at 12,000 × *g* for 10 min. Total viral DNA was extracted using a commercial DNA/RNA extraction kit (Vazyme, Nanjing, China) according to the manufacturer’s instructions. PCV3 DNA was detected using a validated PCR assay (Table S1). Full-length amplification of the viral genome was performed with primers spanning the entire sequence, as previously described (3, 18). The amplified products were purified and cloned into the pMD18-T vector (Takara Bio, Inc., Dalian, China), and the resulting constructs were transformed into competent *Escherichia coli* DH5α cells. Three independent clones from each PCR product were selected for sequencing (Sangon Biotech, Shanghai, China) (3, 19). Consensus sequences were assembled using the Lasergene software suite (DNASTAR Inc., Madison, WI) to reconstruct the complete PCV3 genome.

### Sequence alignment and phylogenetic analyses

The complete genome sequence of the PCV3/SC/Sichuan-2023 strain was deposited in GenBank (accession no. PV700519.1) and compared with 22 reference strains retrieved from the database (Table S2). The coding regions (ORF1 and ORF2) were aligned using the Clustal W algorithm implemented in Lasergene. Genotype classification was performed according to the subtype criteria proposed by Li et al. (20). Phylogenetic analysis was conducted using the maximum-likelihood method in MEGA 7.0, with 1,000 bootstrap replicates applied to evaluate branch support. Amino acid identities of ORF2 sequences were calculated using DNAStar.

### Plasmid construction and viral rescue

The complete PCV3/SC/Sichuan-2023 genome was synthesized and cloned into the pBluescript SK(+) vector at the SalI and EcoRI restriction sites, yielding the recombinant plasmid rPCV3. Sanger sequencing confirmed the absence of unintended nucleotide substitutions. PK-15 cells were seeded in six-well plates and cultured to approximately 70% confluence before transfection with 250 ng of plasmid DNA using Lipofectamine 3000 (Thermo Fisher Scientific, MA, USA). At 4 h post-transfection, the medium was replaced with DMEM supplemented with 2% fetal bovine serum (FBS). Culture supernatants were collected at 72 h post-transfection, clarified by filtration through a 0.22 μm membrane, and subsequently used for serial passage in fresh PK-15 cell cultures. During subsequent infections and serial passages, cells were treated with 300 mM D-glucosamine for 30 min at 24 h post-infection to enhance viral replication, followed by replacement with DMEM containing 2% FBS (21). For each passage, culture supernatants were harvested at 72 h post-infection, including after D-glucosamine treatment, and used for further passages or downstream analyses. Supernatants from passages F0–F12 were collected, and viral DNA copy numbers were quantified by quantitative PCR using a standard curve, as previously described by Deng et al. (22).

### Western blotting

PK-15 cells infected with the F8 virus passage were harvested at 72 h post-infection. After washing twice with cold PBS, the cells were lysed on ice for 20 min using RIPA buffer (Beyotime, Shanghai, China) and clarified by centrifugation at 12,000 rpm for 10 min at 4°C. Proteins were separated by SDS-PAGE, transferred onto PVDF membranes, and immunoblotted with a mouse anti-PCV3 Cap monoclonal antibody (1:500; Qianxun, Guangdong, China) and a horseradish peroxidase-conjugated secondary antibody (1:1,000; Beyotime). Bands were visualized by chemiluminescence.

### Indirect immunofluorescence assay

PK-15 cells infected with the F8-passage virus underwent sequential processing: fixation with 4% paraformaldehyde for 1 h, permeabilization with 0.5% Triton X-100 for 20 min, and blocking with 1% bovine serum albumin (BSA) for 1 h. Immunostaining was performed by overnight incubation at 4°C with the primary antibody anti-PCV3 Cap monoclonal antibody (1:500), followed by a 1 h incubation at 25°C with a fluorescent secondary antibody (FITC-conjugated goat anti-mouse IgG, Beyotime, 1:1,000). After nuclear counterstaining with 4′,6-diamidino-2-phenylindole (DAPI) for 15 min, fluorescence imaging was conducted using a fluorescence microscope.

### Viral growth kinetics

Viral genome copy numbers in infected PK-15 cells were determined at 12, 24, 36, 48, 60, and 72 h post-infection by quantitative PCR using a standard curve. The data were used to generate a viral growth kinetics curve.

### Animal experiments

Eight 28-day-old specific pathogen-free piglets were obtained from Wanjiahao Farm (Meishan, Sichuan) and randomly assigned to two groups, namely: (i) a PCV3-inoculated group and (ii) the uninfected control group. Animals were housed in separate isolation rooms under identical environmental conditions and were provided *ad libitum* access to water and sterilized feed during the acclimation period. Before inoculation, all piglets were screened by real-time PCR for major porcine pathogens, including PCV types 1–4 (PCV1–PCV4), Porcine Epidemic Diarrhea virus, Transmissible Gastroenteritis virus, Pseudorabies virus, PRRSV, and rotavirus, following previously described protocols (23). All tests yielded negative results. As shown in Fig. 3A, piglets in the infection group were inoculated once daily for 3 consecutive days with F8 rPCV3 via intranasal (2 mL per nostril) and intramuscular (1 mL) routes, at a dose of 1 × 10^6.5^ genomic copies per piglet per inoculation. In comparison, control animals received an equivalent volume of sterile PBS via the same routes (15, 24). Clinical signs and rectal temperatures were recorded daily throughout the experimental period. Blood, serum, nasal, oropharyngeal, and rectal swabs were collected at 0, 3, 7, 14, 21, and 28 days post-inoculation (dpi). At 28 dpi, all piglets were humanely euthanized by intravenous administration of sodium pentobarbital (80 mg/kg). Postmortem tissue samples from the liver, heart, lung, spleen, tonsil, kidney, and lymph nodes were collected for viral load determination, histopathological analysis (hematoxylin and eosin [H&E] staining), and immunohistochemical (IHC) examination.

### Histopathological and immunohistochemical analysis

Collected tissues were fixed in 4% paraformaldehyde for 48 h, dehydrated through a graded ethanol series, embedded in paraffin, sectioned at 5 μm thickness, and stained with hematoxylin and eosin (H&E; Solarbio, Shanghai, China) for microscopic evaluation and histological scoring as previously described (24). For IHC analysis, adjacent sections were deparaffinized, rehydrated, and treated with 3% hydrogen peroxide for 10 min to inhibit endogenous peroxidase activity. Nonspecific binding was prevented by incubation with 5% bovine serum albumin (BSA; Thermo Fisher Scientific, USA) for 30 min. Sections were then incubated overnight at 4°C with the primary antibody (anti-PCV3 Cap mAb, 1:200), followed by incubation with a biotinylated secondary antibody and streptavidin–biotin complex, and signals were visualized using 3,3′-diaminobenzidine.

### Serum cytokine analysis by ELISA

For serum preparation, venous blood was collected into sterile plain tubes and allowed to clot for 30 min at 25°C. Samples were then centrifuged at 2,000 × *g* for 15 min at 4°C to obtain the serum fraction. Serum concentrations of interferon-gamma (IFN-γ), tumor necrosis factor-alpha (TNF-α), IL-1β, IL-6, IL-8, IL-12, and IL-10 were measured using commercial porcine enzyme-linked immunosorbent assay (ELISA) kits (Jianglai Biotechnology, Shanghai, China) in accordance with the manufacturer’s instructions. PCV3 Cap protein-specific antibodies were detected using a commercial PCV3 ELISA kit (Koqian Biotechnology, Wuhan, China), with samples considered positive when the *S/P* value was ≥0.15

### Statistical analysis

Data were analyzed using two-way analysis of variance, followed by post hoc multiple-comparison tests. GraphPad Prism version 8.0 was used for statistical analysis and bar graph generation. Results are presented as mean ± standard deviation (SD) from at least three independent replicates. Differences between groups were considered statistically significant at *P* < 0.05.

## RESULTS

### PCV3 detection and genomic analysis

**Fig 1:**
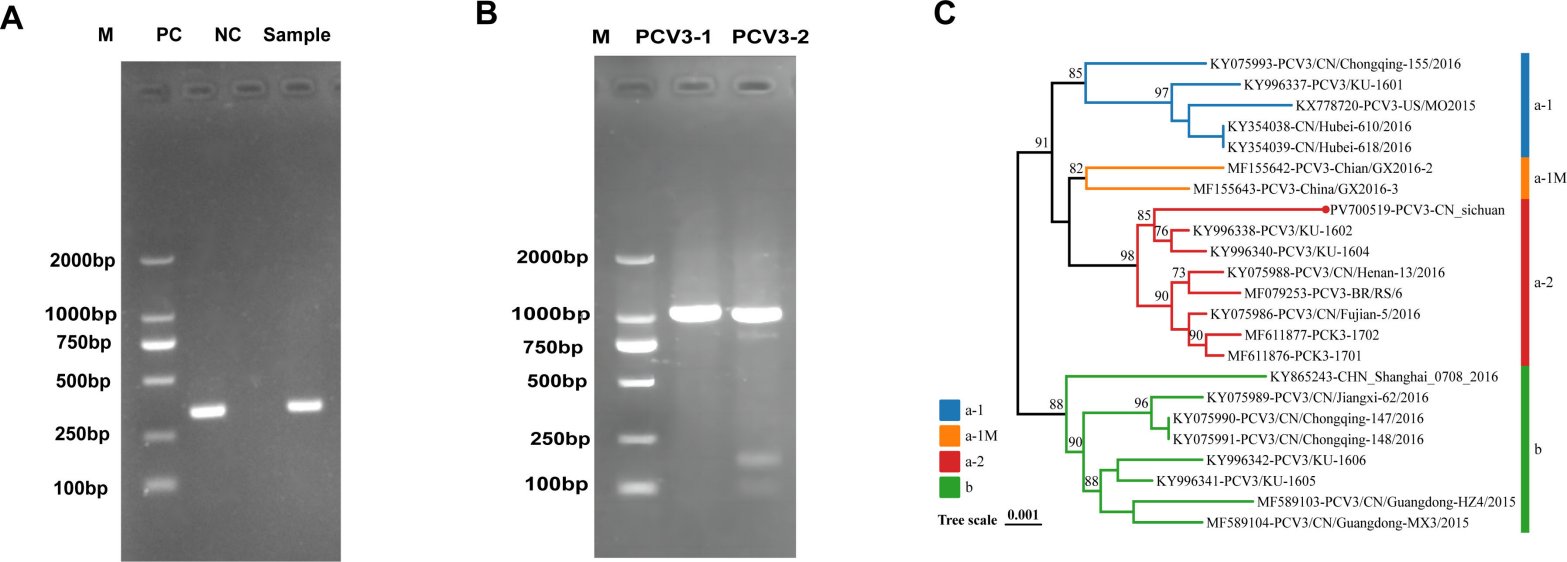
PCR detection, full-length genome amplification, and phylogenetic analysis of PCV3. (**A**) Detection of PCV3-specific fragments in clinical samples. Lane M, DNA marker; Lane PC, positive control; Lane NC, negative control; Lane Sample, clinical sample from a PDNS case. A 344 bp amplicon of the expected size was observed in both the positive control and the clinical sample. (**B**) Amplification of the complete PCV3 genome using two overlapping fragments. Lane M, DNA marker; Lane PCV3-1, 1,240 bp fragment; Lane PCV3-2, 1,017 bp fragment. Both fragments showed clear bands at the expected sizes. (**C**) The bands matched the expected sizes, which confirmed the successful amplification of the full PCV3 genome. Phylogenetic analysis of the ORF1 + ORF2 coding sequences was performed using the neighbor-joining method (p-distance model, 1,000 bootstrap replicates). Strains from this study were labeled with filled circles. Colors represented distinct genotypes.

PCR screening of PDNS-affected tissue samples produced a distinct 344 bp amplicon corresponding to PCV3 (Fig. 1A). Two overlapping fragments (1,240 bp and 1,017 bp) were subsequently amplified and assembled into a complete 2,000 bp genome (Fig. 1B). Phylogenetic analysis of PCV3/SC/Sichuan-2023 (GenBank accession no. PV700519.1) with 23 complete PCV3 genomes retrieved from GenBank (Table S2) classified this isolate within the PCV3a lineage (Fig. 1C). Whole-genome nucleotide identity values ranged from 98.6% to 99.5%, showing the highest similarity (99.5%) to strain PCV3/KU-1602 (Table S3). Three unique amino acid substitutions in the Cap protein (S77T, I150L, and E193G) distinguished this isolate from other strains (Table S4), suggesting possible regional adaptation or host selection pressure.

### Construction and characterization of rescued PCV3

**Fig 2:**
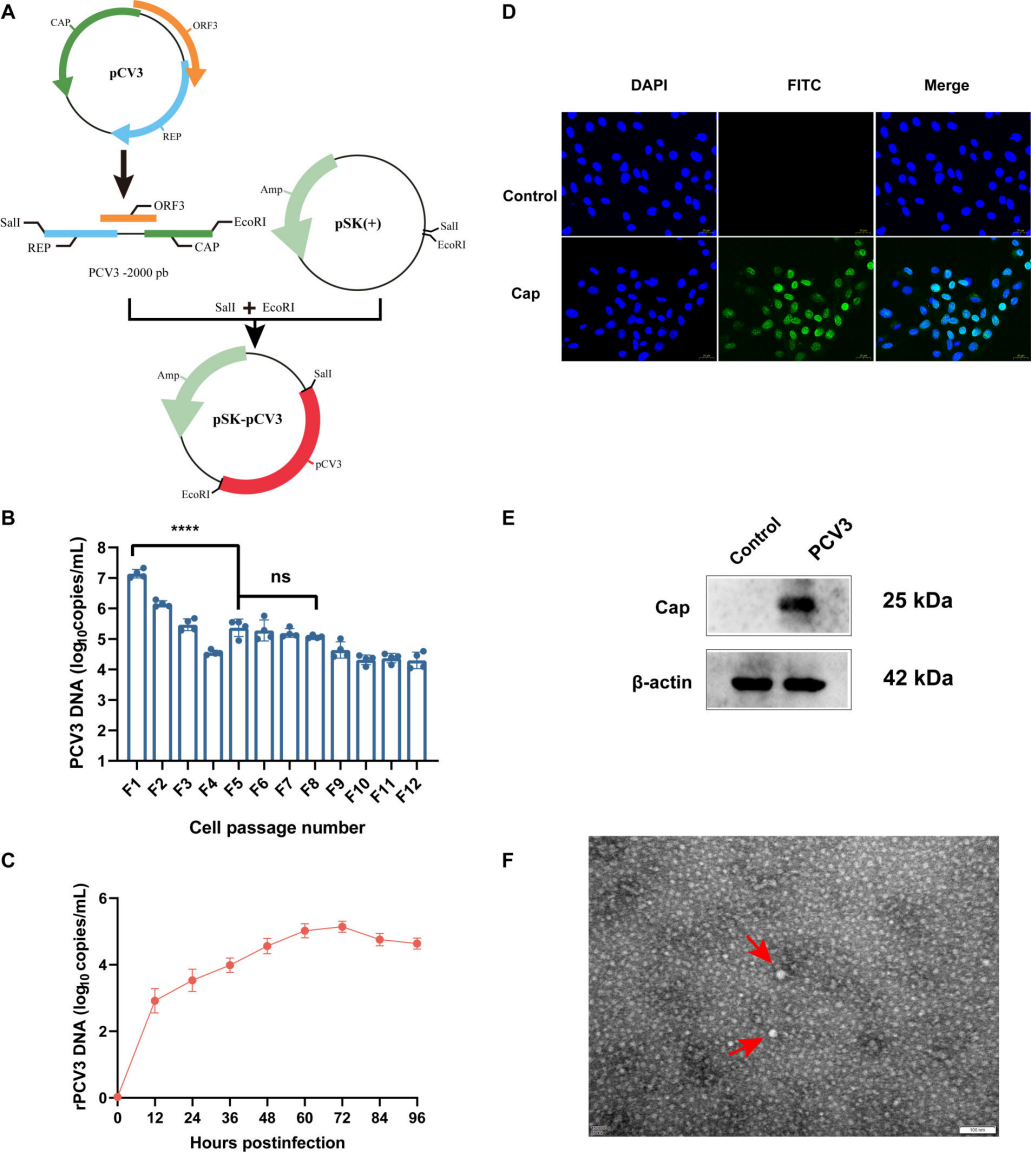
Construction and characterization of the Infectious PCV3 Clone. (**A**) Diagram of the recombinant plasmid pSK-PCV3 construction. The full-length PCV3/Sichuan genome (GenBank: PV700519.1) was synthesized and inserted into the pBluescript SK(+) vector between SalI and EcoRI sites to generate the infectious clone. (**B**) qPCR analysis of viral DNA copy numbers in PK-15 cells at 12 time points. (**C**) qPCR quantification of viral DNA copy numbers at the indicated time points post-infection. (**D**) Immunofluorescence assay detection of PCV3 Cap protein in F8-infected PK-15 cells. The Cap protein (green, FITC) was localized in the cytoplasm, while nuclei were counterstained with DAPI (blue). No signal was seen in the uninfected control. (**E**) Western blot analysis of PCV3 Cap protein expression in F8-infected PK-15 cells. A specific band at approximately 25 kDa was detected in infected cells but not in the control. β-Actin (42 kDa) served as the loading control. (**F**) Transmission electron microscopy revealed spherical virus-like particles approximately 20 nm in diameter in PCV3-infected samples (red arrows). Scale bar: 100 nm. Data are presented as mean ± SD. Statistical significance: ns, not significant; ****P < 0.0001.

To evaluate the infectivity of the Sichuan isolate, a full-length infectious clone (rPCV3) was constructed by inserting the PCV3/CN/Sichuan-2023 genome into the pBluescript SK(+) vector at the SalI and EcoRI restriction sites (Fig. 2A). Sanger sequencing confirmed complete sequence integrity, with no unintended mutations detected. PK-15 cells were transfected with the recombinant plasmid (pSK-PCV3) using Lipofectamine 3000. At 72 h post-transfection, DNase I-treated culture supernatants were serially passaged in PK-15 cells up to passage F12. Quantitative PCR detected PCV3 genomic DNA across all passages, demonstrating successful virus rescue and sustained replication *in vitro* (Fig. 2B). Following rPCV3 infection of PK-15 cells, viral genome copy numbers were quantified by quantitative PCR at the indicated time points. Viral replication increased during the early phase of infection, reached peak levels at approximately 60–72 h post-infection, and subsequently plateaued or declined slightly at later time points (Fig. 2C). To confirm viral protein expression, indirect immunofluorescence analysis was performed on PK-15 cells infected with the F8 passage. Strong cytoplasmic fluorescence corresponding to PCV3 Cap protein was observed in infected cells, whereas no signal was detected in mock-infected controls (Fig. 2D). Western blot analysis further verified Cap protein expression, revealing a distinct ~25 kDa band in lysates from F8-infected cells, absent in control samples (Fig. 2E). Transmission electron microscopy identified spherical, icosahedral viral particles approximately 20 nm in diameter, consistent with the morphology of native PCV3 virions (Fig. 2F). These results confirm the successful rescue of an infectious PCV3 clone and its stable propagation in cell culture.

### Clinical manifestations in rPCV3-infected piglets

**Fig 3:**
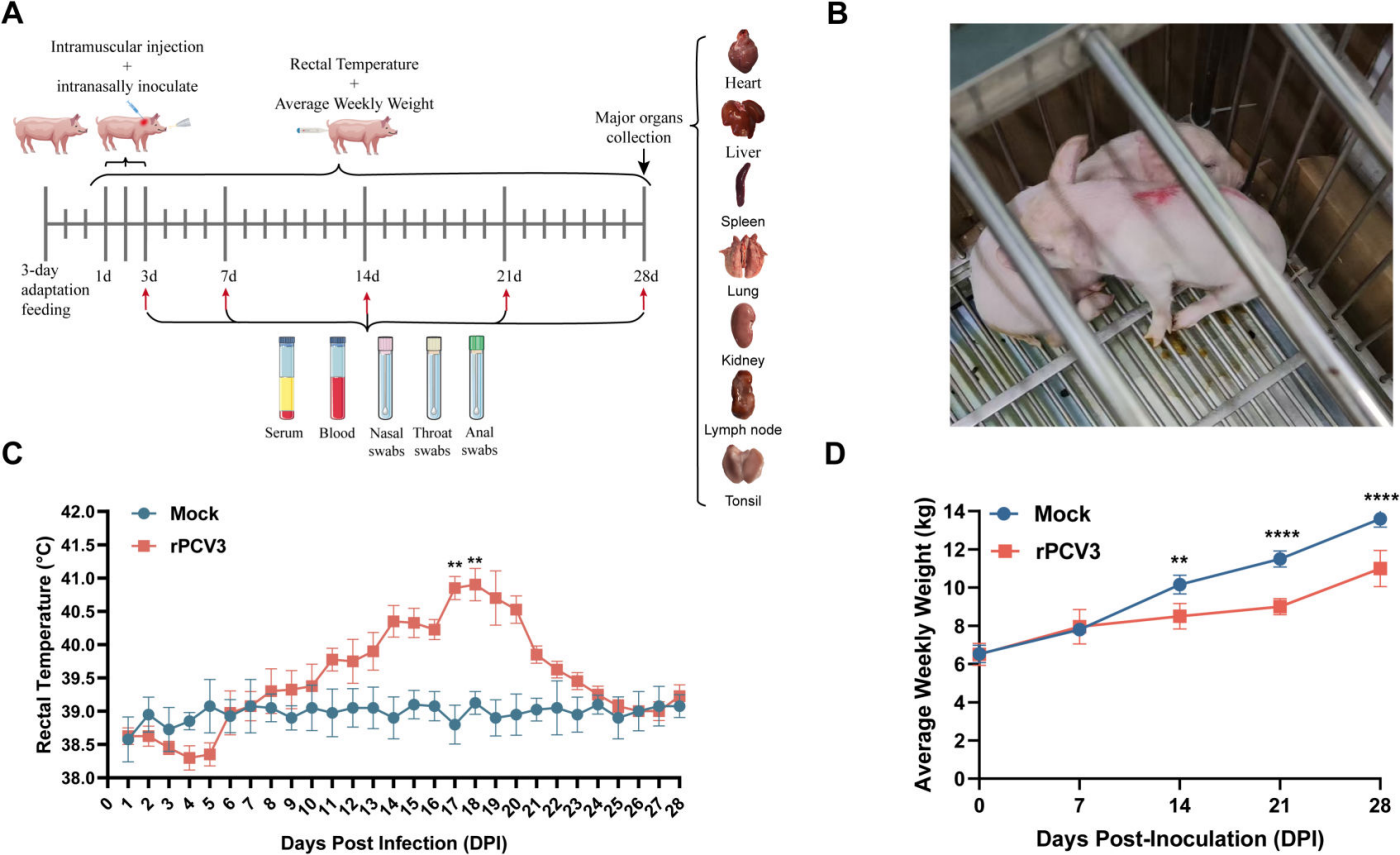
Clinical manifestations of piglets following rPCV3 infection. (**A**) Schematic diagram of the experimental design and inoculation protocol. (**B**) Clinical photograph of diarrhea in a PCV3-infected piglet. (**C**) Daily rectal temperature measurements of piglets post-infection. (**D**) Weekly changes in body weight of piglets following infection. Data were presented as mean ± SD. Statistical significance: ns, not significant; **P < 0.01; ****P < 0.0001.

After a 3-day acclimation period, piglets were inoculated with rPCV3 via intramuscular and intranasal routes; clinical signs were monitored, and samples were collected throughout the experiment (Fig. 3A). Piglets inoculated with rPCV3 began to exhibit mild but discernible clinical changes around 11 dpi, primarily characterized by reduced feed intake and transient diarrhea (Fig. 3B). Daily monitoring revealed a gradual rise in rectal temperature starting at 6 dpi, peaking between 16 dpi and 18 dpi (Fig. 3C). Weekly weight measurements showed a significant reduction in body weight gain from 14 dpi onward compared with PBS-treated controls (Fig. 3D). All control piglets remained clinically healthy throughout the experimental period.

### Viral load dynamics following rPCV3 infection

**Fig 4:**
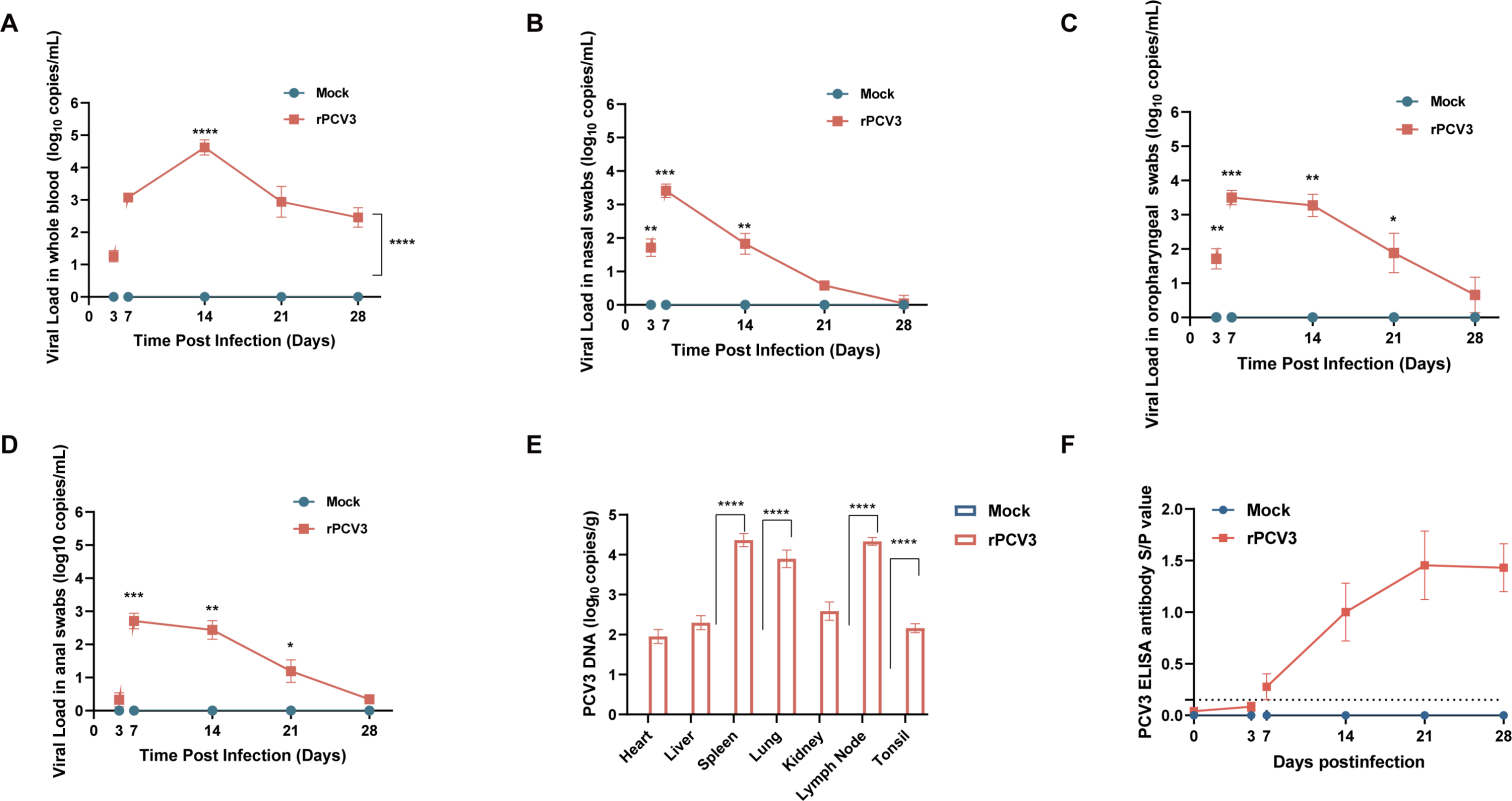
Overall virological and serological responses in PCV3-infected piglets. (**A**) Viremia detected in serum. (**B**) Viral shedding detected in nasal swabs. (**C**) Viral shedding detected in oropharyngeal swabs. (**D**) Viral shedding detected in anal swabs. (**E**) Quantification of viral loads in different organs. (**F**) Detection of PCV3 antibodies in piglet serum. PCV3 Cap protein-specific antibodies were measured by ELISA. An *S*/*P* ratio ≥ 0.15 was considered evidence of seropositivity. Data were presented as mean ± SD. Statistical significance: ns, not significant; *P < 0.05; **P < 0.01; ***P < 0.001; ****P < 0.0001.

Throughout the study, PCV3 DNA was undetectable in serum and swab samples from the control group. In contrast, rPCV3-infected piglets showed evidence of systemic viral replication, with detectable viremia and viral shedding at mucosal sites. Viral DNA was first observed in serum at 3 dpi and peaked at 21 dpi (Fig. 4A). Similarly, viral loads in nasal, oropharyngeal, and anal swabs increased substantially from 3 dpi and peaked at 7 dpi (Fig. 4B through D). Quantitative PCR of tissue samples revealed the spleen, lymph nodes, tonsils, and lungs as the major sites of viral accumulation (Fig. 4E), indicating that rPCV3 displays broad tissue tropism. Serological analysis showed that PCV3 Cap protein-specific antibodies became detectable in rPCV3-infected piglets at 7 dpi, with *S*/*P* values exceeding the cut-off. Antibody levels increased thereafter and reached the highest levels at 21 dpi, followed by a slight decline at 28 dpi. In contrast, mock-infected piglets remained seronegative throughout the observation period (Fig. 4F).

### Histopathological characterization of lesions associated with PCV3 infection

**Fig 5:**
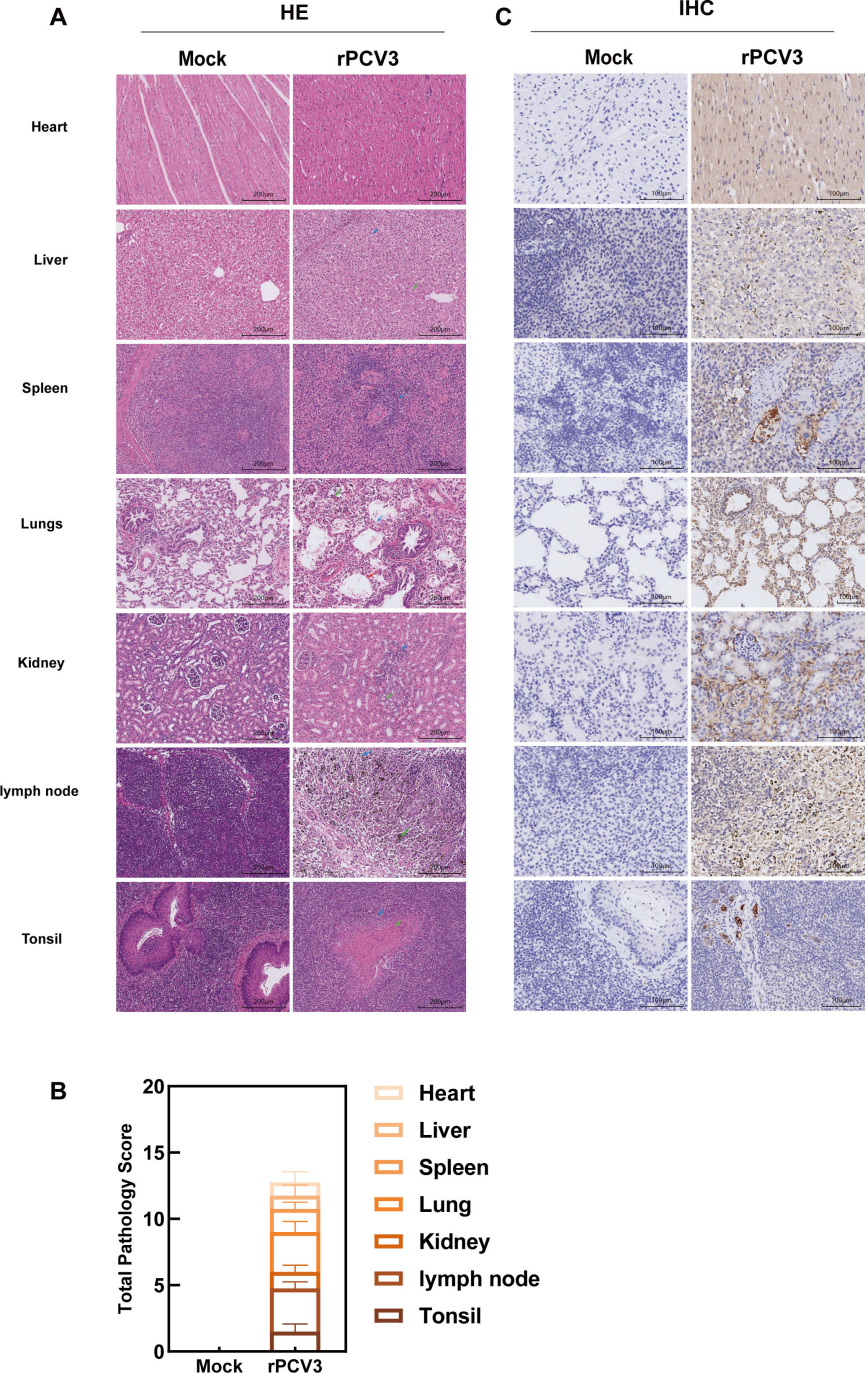
Histopathology and IHC of tissues from PCV3-infected piglets. (**A**) H&E-stained sections of the heart, liver, spleen, lungs, kidneys, lymph nodes, and tonsils from mock and rPCV3 piglets at 28 dpi (100×; scale bars, 200 µm). Representative pathological changes were indicated by arrows. (**B**) Total pathology scores of the major organs in mock and rPCV3-infected piglets. Lesions in the heart, liver, spleen, lungs, kidneys, lymph nodes, and tonsils were scored as described. (**C**) IHC for PCV3 Cap in the same tissues at 28 dpi (200×; scale bars, 100 µm). Brown staining indicates Cap-positive signals.

Microscopic examination revealed characteristic histopathological alterations in multiple organs of rPCV3-infected piglets (Fig. 5A). In the spleen, the white pulp was reduced, accompanied by decreased lymphocyte density (green arrow). Lung sections showed extensive necrosis and sloughing of the bronchial epithelium (green arrow), disruption of alveolar architecture with mild inflammatory cell infiltration (blue arrow), and focal alveolar dilation in adjacent areas (red arrow). The kidneys demonstrated mild tubular edema at the corticomedullary junction (black arrow), focal tubular atrophy (green arrow), increased epithelial basophilia with luminal narrowing, and mild peritubular inflammatory infiltration (blue arrow). Lymphoid tissues displayed focal necrosis (green arrow) and lymphocyte depletion (blue arrow). Tonsillar sections demonstrated densely packed lymphoid follicles and uniformly distributed crypts, along with mild epithelial infiltration (green arrow) and crypt lumens containing inflammatory exudates (blue arrow). These lesions corresponded with viral load distribution, confirming that PCV3 infection induces multisystem pathological injury.

Pathological changes in individual tissues were evaluated using a four-grade lesion scoring system (25). Overall pathological scores were significantly higher in the rPCV3-infected group than in the mock control group (Fig. 5B). In infected piglets, lesions of varying severity were observed across multiple organs, with more pronounced changes in the lungs, lymph nodes, and tonsils. In comparison, the kidneys, spleen, and liver demonstrated mild-to-moderate alterations. In comparison, tissues from control animals showed no apparent pathological abnormalities. These results indicate that rPCV3 infection results in multisystem pathological damage *in vivo*.

### PCV3 antigen tissue distribution analysis

Immunohistochemical analysis demonstrated widespread distribution of PCV3 antigens across multiple organs (Fig. 5B). In cardiac tissue, myocardial fibers were well organized with elongated, spindle-shaped nuclei, and viral antigens were predominantly localized within cardiomyocyte nuclei. Hepatic sections revealed antigen staining in both hepatocytes and sinusoidal endothelial cells, frequently accompanied by focal inflammatory infiltrates. In the spleen, the demarcation between red and white pulp was indistinct and associated with vascular hyalinization, lymphocytic infiltration, and cytoplasmic antigen deposition in subsets of lymphocytes. In lung tissue, PCV3 antigen was detected in both the cytoplasm and nuclei of alveolar and bronchial epithelial cells. Renal sections showed largely intact glomeruli and tubules, although sporadic interstitial cells demonstrated cytoplasmic antigen positivity. Lymph nodes displayed disrupted corticomedullary architecture with occasional residual follicles and positive antigen staining in both paracortical and medullary regions. Tonsillar sections demonstrated crypt epithelial desquamation and follicular hyperplasia, with viral antigens distributed throughout the fibrous stroma and lymphoid aggregates. These observations indicate that PCV3 presents broad tissue tropism, with a preference for lymphoid and parenchymal organs.

### Cytokine responses to PCV3 infection

**Fig 6:**
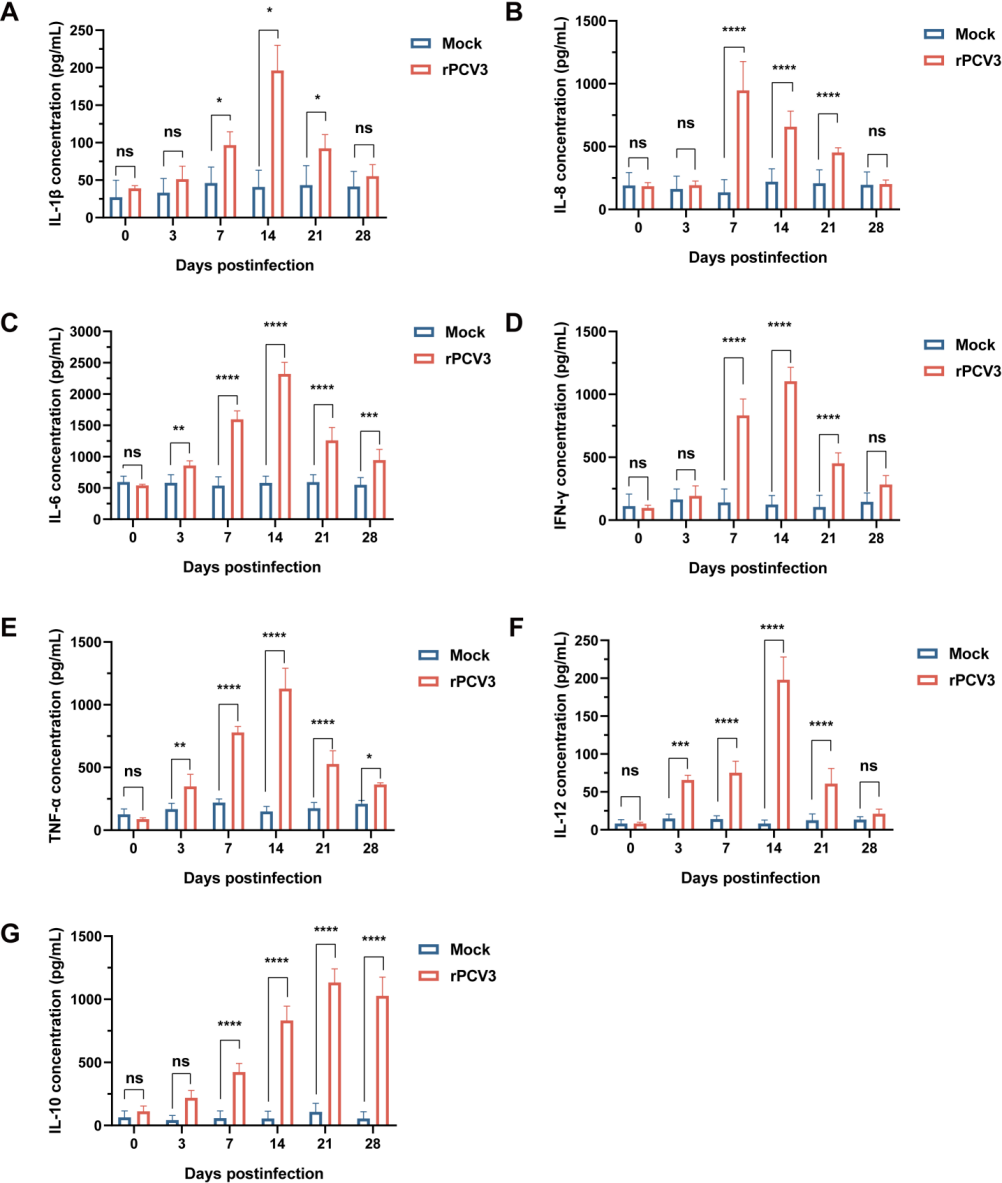
Cytokine levels in the serum of piglets. Cytokine levels of IL-1β (**A**), IL-8 (**B**), IL-6 (**C**), IFN-γ (**D**), TNF-α (**E**), IL-12 (**F**), and IL-10 (**G**) were measured using ELISA. Data were presented as mean ± SD. Statistical significance: ns, not significant; *P < 0.05; **P < 0.01; ***P < 0.001; ****P < 0.0001.

Infection with rPCV3 induced a robust and dynamic cytokine response in piglets (Fig. 6A through G). Serum concentrations of the pro-inflammatory cytokines IL-1β, IL-6, IL-8, TNF-α, IFN-γ, and IL-12, as well as the anti-inflammatory cytokine IL-10, were significantly elevated in the rPCV3-infected group compared with mock controls at multiple time points post-infection. IL-1β showed a transient increase, peaking at 14 dpi and gradually declining thereafter (Fig. 6A). IL-8 levels rose sharply during the acute phase at 7 dpi, decreased by 14 dpi, and returned to near-baseline values by 28 dpi (Fig. 6B). IL-6, IFN-γ, and TNF-α demonstrated sustained elevations beginning at 7 dpi, reaching maximal levels at 14 dpi, followed by a progressive decline toward baseline by 28 dpi (Fig. 6C through E). In comparison, IL-12 displayed a delayed kinetic profile, gradually increasing from 3 to 7 dpi, peaking at 14 dpi, and subsequently decreasing (Fig. 6F). The anti-inflammatory cytokine IL-10 showed a progressive increase, with peak levels observed at 7 and 14 dpi (Fig. 6G). These findings indicate that rPCV3 infection triggers a pronounced yet transient pro-inflammatory cytokine cascade dominated by IL-1β, IL-6, IL-8, TNF-α, and IFN-γ during the acute phase, followed by a compensatory rise in IL-10 during the recovery period.

## DISCUSSION

Since its initial identification in 2016, PCV3 has been reported in swine populations worldwide (26–28). Although it is now recognized as a clinically relevant pathogen, the molecular basis of its pathogenicity remains incompletely understood, particularly for strains circulating in southwestern China. In this study, we successfully obtained the complete genome of PCV3/SC/Sichuan-2023 from piglets presenting with rough hair coats and multifocal papular lesions. Phylogenetic analysis assigned this isolate to the PCV3a lineage, consistent with the predominant genotypes currently circulating in the region (17). Comparative genomic analysis revealed 99.5% nucleotide identity with PCV3/KU-1602 (South Korea, 2016), highlighting the high level of genomic conservation among PCV3 strains across geographic regions. This degree of conservation supports the widespread dissemination of closely related lineages and underscores the importance of continued molecular surveillance. Three amino acid substitutions were identified in the Cap protein (S77T, I150L, and E193G). Among these, E193G is located within the C-terminal region, whereas S77T and I150L involve residues with similar physicochemical properties. Although the biological and immunological implications of these substitutions remain unclear, they provide valuable sequence information for future functional and antigenic studies. To facilitate downstream investigations, we established a full-length infectious clone (rPCV3) that enabled virus rescue, stable replication, and consistent Cap protein expression in PK-15 cells. This system complements previous PCV3 isolation efforts reported by Mora-Díaz et al. (29) and Oh and Chae (30). Given that PCV2 and PCV3 share only approximately 48% genomic identity, existing PCV2-based vaccines are unlikely to confer effective cross-protection (31, 32). Therefore, the rescued rPCV3 strain represents a practical experimental platform for dissecting PCV3 pathogenesis and supporting the development of PCV3-specific vaccines.

Compared with earlier PCV3 rescue-and-challenge models (33), our rPCV3 system achieved robust viral replication and reproducible antigen expression *in vivo*. The dual-route inoculation strategy, combining intramuscular and intranasal administration over 3 consecutive days (34), effectively mimicked chronic, low-grade exposure under field conditions. This approach reproduced the gradual onset of clinical signs, persistent viremia, and multifocal lesions characteristic of natural PCV3 infection. Although PDNS was not observed in this controlled setting, unlike the findings of Jiang et al. (15), it is likely that PDNS development requires synergistic effects of environmental stressors or co-infections that are absent under laboratory conditions (2). These observations highlight the multifactorial nature of PCV3-associated disease and emphasize the importance of investigating infection dynamics within broader ecological contexts. To further characterize viral dissemination, we analyzed shedding patterns and tissue distribution of this PCV3a strain. Quantitative analyses of nasal, oropharyngeal, and rectal swabs demonstrated efficient replication in upper respiratory tissues followed by systemic spread via the bloodstream. Compared with previous studies reporting relatively mild infection (15), this isolate showed broader tissue tropism and more pronounced pathology, with high viral loads detected in the spleen, lungs, lymph nodes, and tonsils, accompanied by significant histopathological changes. These findings are consistent with earlier reports (35, 36) but indicate an even more extensive dissemination profile. The temporal accumulation of virus in pulmonary tissue and subsequent enrichment in lymphoid organs suggests dynamic interactions with immune cells, potentially facilitating viral persistence through immune modulation. Detection of viral antigens in both the cytoplasm and the nucleus of lymphocytes further supports the possibility of latency or immune evasion.

Finally, cytokine profiling provided additional insight into host immune responses following PCV3 infection. Cytokines coordinate innate and adaptive immunity, and their temporal patterns reflect the balance between host defense and immune regulation. We quantified IL-1β, IL-6, IL-8, IFN-γ, TNF-α, IL-12, and IL-10 to delineate the kinetics of immune activation. Early elevation of IL-1β indicated acute inflammatory responses, followed by sustained increases in IL-6 and TNF-α that coincided with peak viral replication in the lungs and lymph nodes. Elevated IFN-γ levels reflected activation of T cells and natural killer cells, which are critical for antiviral defense. Progressive increases in IL-10 suggest activation of anti-inflammatory feedback mechanisms to restrain excessive immune activation, while IL-12 upregulation indicates enhanced T-cell stimulation (15, 37). Our data reveal a sequential immune profile encompassing initial pro-inflammatory activation, subsequent antiviral responses, and late-stage immune regulation. This progression underscores the complementary roles of IL-10 and IL-12 in balancing inflammation and adaptive immunity, offering new insights into how PCV3 may manipulate host immune responses to establish persistence. Given the increasing globalization of swine production, continuous transboundary surveillance of PCV3 remains essential for effective disease monitoring and control.

### Conclusion

In conclusion, this study successfully rescued and characterized a clinically relevant PCV3 strain, demonstrating efficient viral replication both *in vitro* and *in vivo*. The virus induced pronounced lymphoid tissue injury in piglets, highlighting its pathogenic potential. The optimized strain selection and inoculation strategy establish a robust experimental framework for future studies of PCV3 pathogenesis, immune evasion, and vaccine development. These findings advance current understanding of PCV3 biology and provide a solid foundation for the development of targeted therapeutic and preventive approaches. Given the increasing globalization of swine production, sustained surveillance of PCV3 is essential to mitigate the risk of transboundary transmission and to support effective disease control.

## Data Availability

The genome sequences identified in this study have been deposited in the GenBank database. The complete genome sequence of the PCV3/SC/Sichuan/2023 isolate has been assigned the accession number PV700519.1. The data that support the findings of this study were available from the corresponding author upon reasonable request.

## SUPPLEMENTAL MATERIAL

Supplemental material spectrum.03865-25-s0001.docx Tables S1 to S4; Fig. S1.

ASM does not own the copyrights to Supplemental Material that may be linked to, or accessed through, an article. The authors have granted ASM a non-exclusive, world-wide license to publish the Supplemental Material files. Please contact the corresponding author directly for reuse.
