# Supplementary material for: Construction and in vivo characterization of an infectious clone of porcine circovirus type 3 from southwestern China
Source: Microbiol Spectr. 2026 Apr 13;14(5):e03865-25. doi: 10.1128/spectrum.03865-25 (PMC13142002; doi:10.1128/spectrum.03865-25)
Supplement: Supplemental material — Tables S1 to S4; Fig. S1. [file spectrum.03865-25-s0001.docx]

**Supplementary Table and Figure**

**Supplementary** **Table 1** Primers for PCV3 detection and full-length genome amplification.

| Primer Name | Primer Sequence | Length/bp |
| --- | --- | --- |
| PCV3-F | CGACCGAGTGGGAATCTA | 344 |
| PCV3-R | AGGCATCTTCTCCGCAAC |  |
| PCV3-1-F | TCGTG GAAAGTTGGAGGC | 1240 |
| PCV3-1-R | AGTCCTTATCTTCAGGACACTCG |  |
| PCV3-2-F | CGGATCCACGGAGGTCT | 1017 |
| PCV3-2-R | GTGCGGGCACAGGTAAAC |  |

**Supplementary** **Table 2** Reference strains used in the phylogenetic analysis for this study.

| **Number** | **Reference strain** | **Origin** | **Isolation year** | **GenBank accession number** |
| --- | --- | --- | --- | --- |
| 1 | PCV3-US/MO2015 | USA | 2015 | KX778720 |
| 2 | PCV3/CN/Chongqing-155/2016 | China | 2016 | KY075993 |
| 3 | CN/Hubei-610/2016 | China | 2016 | KY354038 |
| 4 | CN/Hubei-618/2016 | China | 2016 | KY354039 |
| 5 | PCV3/KU-1601 | South Korea | 2016 | KY996337 |
| 6 | PCV3-Chian/GX2016-2 | China | 2016 | MF155642 |
| 7 | PCV3-China/GX2016-3 | China | 2016 | MF155643 |
| 8 | PCV3/CN/Fujian-5/2016 | China | 2016 | KY075986 |
| 9 | PCV3-CN-Henan-13-2016 | China | 2016 | KY075988 |
| 10 | PCV3/KU-1602 | South Korea | 2016 | KY996338 |
| 11 | PCV3/KU-1604 | South Korea | 2016 | KY996340 |
| 12 | PCV3-BR/RS/6 | Brazil | 2016 | MF079253 |
| 13 | PCK3-1701 | South Korea | 2016 | MF611876 |
| 14 | PCK3-1702 | South Korea | 2016 | MF611877 |
| 15 | PCV3/CN/Jiangxi-62/2016 | China | 2016 | KY075989 |
| 16 | PCV3/CN/Chongqing-147/2016 | China | 2016 | KY075990 |
| 17 | PCV3/CN/Chongqing-148/2016 | China | 2016 | KY075991 |
| 18 | CHN_Shanghai_0708_2016 | China | 2016 | KY865243 |

**Supplementary** **Table 2** (continued).

| **Number** | **Reference strain** | **Origin** | **Isolation year** | **GenBank accession number** |
| --- | --- | --- | --- | --- |
| 19 | PCV3/KU-1605 | South Korea | 2016 | KY996341 |
| 20 | PCV3/KU-1606 | South Korea | 2016 | KY996342 |
| 21 | PCV3/CN/Guangdong-HZ4/2015 | China | 2015 | MF589103 |
| 22 | PCV3/CN/Guangdong-MX3/2015 | China | 2015 | MF589104 |

**Supplementary** **Table 3** Nucleotide sequence identity of PCV3 strains.

| **Number** | **Reference strain** | **GenBank accession number** | **Complete genome identity (%)** | **Cap gene identity (%)** |
| --- | --- | --- | --- | --- |
| 1 | PCV3-US/MO2015 | KX778720 | 98.6 | 98.1 |
| 2 | PCV3/CN/Chongqing-155/2016 | KY075993 | 98.9 | 98.6 |
| 3 | CN/Hubei-610/2016 | KY354038 | 98.8 | 98.1 |
| 4 | CN/Hubei-618/2016 | KY354039 | 98.8 | 98.1 |
| 5 | PCV3/KU-1601 | KY996337 | 98.7 | 98.0 |
| 6 | PCV3-Chian/GX2016-2 | MF155642 | 98.9 | 98.4 |
| 7 | PCV3-China/GX2016-3 | MF155643 | 98.9 | 98.6 |
| 8 | PCV3/CN/Fujian-5/2016 | KY075986 | 99.3 | 99.5 |
| 9 | PCV3-CN-Henan-13-2016 | KY075988 | 99.2 | 99.4 |
| 10 | PCV3/KU-1602 | KY996338 | 99.5 | 99.7 |
| 11 | PCV3/KU-1604 | KY996340 | 99.4 | 99.5 |
| 12 | PCV3-BR/RS/6 | MF079253 | 99.2 | 99.4 |
| 13 | PCK3-1701 | MF611876 | 99.3 | 99.5 |
| 14 | PCK3-1702 | MF611877 | 99.2 | 99.2 |
| 15 | PCV3/CN/Jiangxi-62/2016 | KY075989 | 98.6 | 98.1 |
| 16 | PCV3/CN/Chongqing-147/2016 | KY075990 | 98.7 | 98.3 |
| 17 | PCV3/CN/Chongqing-148/2016 | KY075991 | 98.7 | 98.3 |
| 18 | CHN_Shanghai_0708_2016 | KY865243 | 98.6 | 98.0 |
| 19 | PCV3/KU-1605 | KY996341 | 98.9 | 98.6 |

**Supplementary** **Table 3** (continued).

| **Number** | **Reference strain** | **GenBank accession number** | **Complete genome identity (%)** | **Cap gene identity (%)** |
| --- | --- | --- | --- | --- |
| 20 | PCV3/KU-1606 | KY996342 | 98.7 | 98.3 |
| 21 | PCV3/CN/Guangdong-HZ4/2015 | MF589103 | 98.6 | 98.1 |
| 22 | PCV3/CN/Guangdong-MX3/2015 | MF589104 | 98.8 | 98.3 |

**Supplementary** **Table 4** Amino acid substitutions in the Cap protein among PCV3 strains.

| **Number** | **Reference strain** | | | | | | | | | |
| --- | --- | --- | --- | --- | --- | --- | --- | --- | --- | --- |
|  | **10** | **20** | **24** | **27** | **77** | **98** | **104** | **132** | **150** | **193** |
| KX778720-PCV3-US/MO2015 | R | R | A | R | S | Q | F | R | I | E |
| PV700519-PCV3-CN sichuan | . | . | . | . | T | . | . | . | L | G |
| KY075986-PCV3/CN/Fujian-5/2016 | . | . | . | . | T | . | . | . | L | . |
| KY075988-PCV3/CN/Henan-13/2016 | . | . | . | . | T | . | . | . | L | . |
| KY075989-PCV3/CN/Jiangxi-62/2016 | . | . | V | K | . | . | . | . | . | . |
| KY075990-PCV3/CN/Chongqing-147/2016 | . | . | V | K | . | . | . | . | . | . |
| KY075991-PCV3/CN/Chongqing-148/2016 | . | . | V | K | . | . | . | . | . | . |
| KY075993-PCV3/CN/Chongqing-155/2016 | . | . | . | . | . | . | . | . | . | . |
| KY354038-CN/Hubei-610/2016 | . | . | . | . | . | . | . | . | . | . |
| KY354039-CN/Hubei-618/2016 | . | . | . | . | . | . | . | . | . | . |
| KY865243-CHN Shanghai 0708 2016 | . | . | V | K | N | . | . | . | . | . |
| KY996337-PCV3/KU-1601 | . | . | V | . | . | . | . | . | . | . |
| KY996338-PCV3/KU-1602 | . | . | . | . | T | . | . | . | L | . |
| KY996340-PCV3/KU-1604 | . | . | . | . | T | . | . | . | L | . |
| KY996341-PCV3/KU-1605 | . | . | V | K | . | . | . | . | . | . |
| KY996342-PCV3/KU-1606 | . | K | V | K | . | . | . | . | . | . |
| MF079253-PCV3-BR/RS/6 | . | . | . | . | T | . | . | . | L | . |
| MF155642-PCV3-Chian/GX2016-2 | K | . | . | . | . | . | Y | . | . | . |

**Supplementary** **Table 4** (continued).

| **Number** | **Reference strain** | | | | | | | | | |
| --- | --- | --- | --- | --- | --- | --- | --- | --- | --- | --- |
| MF155643-PCV3-China/GX2016-3 | . | . | . | . | . | . | . | . | . | . |
| MF589103-PCV3/CN/Guangdong-HZ4/2015 | . | . | V | K | . | K | . | . | . | . |
| MF589104-PCV3/CN/Guangdong-MX/2015 | . | . | V | K | . | . | . | . | . | . |
| MF611876-PCK3-1701 | . | . | . | . | T | . | . | . | L | . |
| MF611877-PCK3-1702 | . | . | . | . | T | . | . | G | L | . |


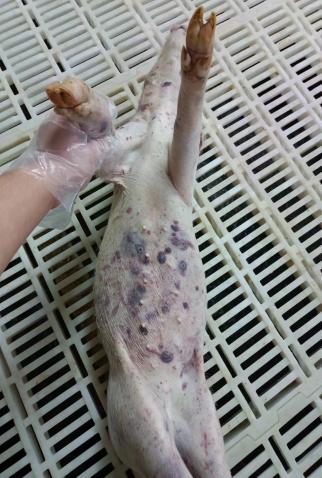


**Supplementary Figure 1** Clinical symptoms in piglets infected with PCV3: The skin exhibited rough texture, with multifocal papules and macules.
